# Wild plant folk nomenclature of the Mongol herdsmen in the Arhorchin national nature reserve, Inner Mongolia, PR China

**DOI:** 10.1186/1746-4269-9-30

**Published:** 2013-04-24

**Authors:** Soyolt, Galsannorbu, Yongping, Wunenbayar, Guohou Liu, Khasbagan

**Affiliations:** 1 Ecology and Environment College, Inner Mongolia Agricultural University, Hohhot 010019, PR China; 2 College of Life Science and Technology, Inner Mongolia Normal University, Hohhot 010022, PR China; 3 Administration Bureau for Arhorchin National Nature Reserve in Inner Mongolia, Arhorchin 025550, PR China

**Keywords:** Wild plants, The Mongol herdsmen, Folk nomenclature, Arhorchin National Nature Reserve, Inner Mongolia

## Abstract

**Background:**

Folk names of plants are the root of traditional plant biodiversity knowledge. In pace with social change and economic development, Mongolian knowledge concerning plant diversity is gradually vanishing. Collection and analysis of Mongolian folk names of plants is extremely important. During 2008 to 2012, the authors have been to the Arhorchin National Nature Reserve area 5 times. Fieldwork was done in 13 villages, with 56 local Mongol herdsmen being interviewed. This report documents plant folk names, analyzes the relationship between folk names and scientific names, looks at the structure and special characteristics of folk names, plant use information, and comparative analysis were also improved.

**Methods:**

Ethnobotanical interviewing methods of free-listing and open-ended questionnaires were used. Ethnobotanical interview and voucher specimen collection were carried out in two ways as local plant specimens were collected beforehand and then used in interviews, and local Mongol herdsmen were invited to the field and interviewed while collecting voucher specimens. Mongolian oral language was used as the working language and findings were originally recorded in Mongolian written language. Scientific names of plants are defined through collection and identification of voucher specimens by the methods of plant taxonomy.

**Results:**

A total of 146 folk names of local plants are recorded. Plant folk names corresponded with 111 species, 1 subspecies, 7 varieties, 1 form, which belong to 42 families and 88 genera. The correspondence between plant folk names and scientific names may be classified as one to one correspondence, two or three to one correspondence, and one to multitude correspondence. The structure of folk names were classified as primary names, secondary names and borrowed names. There were 12 folk names that contain animal names and they have correspondence with 15 species. There are nine folk names that contain usage information and they have correspondence with 10 species in which five species and one variety of plant are still used by the local people. The results of comparative analysis on the Mongol herdsmen in the Arhorchin National Nature Reserve and the Mongolians in the Ejina desert area shows that there are some similarities, as well as many differences whether in language or in the structure.

**Conclusion:**

In the corresponding rate between plant folk names and scientific names yielded a computational correspondence of 82.19%, which can be considered as a high level of consistency between scientific knowledge and traditional knowledge in botanical nomenclature. Primary names have most cultural significance in the plant folk names. Special characteristic of plant folk names were focused on the physical characteristics of animals which were closely related to their traditional animal husbandry and environment. Plant folk names are not only a code to distinguish between different plant species, but also a kind of culture rich in a deep knowledge concerning nature. The results of comparative analysis shows that Mongolian culture in terms of plant nomenclature have characteristics of diversity between the different regions and different tribes.

## Background

Article 8 (j) of the "Convention on Biological Diversity" (CBD) describes that subject to its national legislation, respect, preserve and maintain knowledge, innovations and practices of indigenous and local communities embodying traditional lifestyles relevant for the conservation and sustainable use of biological diversity and promote their wider application with the approval and involvement of the holders of such knowledge, innovations and practices and encourage the equitable sharing of the benefits arising from the utilization of such knowledge, innovations and practices [1]. Article 2 (Definitions) of "The Convention for the Safeguarding of Intangible Cultural Heritage" (ICH) describes that the intangible cultural heritage means the practices, representations, expressions, knowledge, skills – as well as the instruments, objects, artifacts and cultural spaces associated therewith – that communities, groups and, in some cases, individuals recognize as part of their cultural heritage [2]. “Oral traditions and expressions, including language as a vehicle of the intangible cultural heritage” and “knowledge and practices concerning nature and the universe” were parts of the domains of ICH.

Folk names of plants are the root of traditional plant biodiversity knowledge. Indigenous knowledge is the systematic information that remains in diverse social structures. It is usually unwritten and preserved only through oral tradition, and it refers to the knowledge system of indigenous people and minority cultures. Traditional knowledge of biodiversity concerns the names, uses, and management of plants and animals as perceived by the local and or indigenous people of a given area. In the ethnobiological and anthropological area, Berlin has indicated a strong need for linking the scientific and folk systems of classification [3]. Examples of such links have been quoted by Berlin et al. who has looked at the relationship between folk names and scientific names [4-7]. The earliest research on plant folk nomenclature of minority nationalities of China was launched in Xishuangbanna which aims were to study the plant folk nomenclature and taxonomic system of Dai nationality [8], and folk nomenclature of rattan by Hani nationality [9]. These works have laid the foundation for folk taxonomical study of the minority nationalities in China.

In pace with social change and development, the Mongols are changing from nomadic people into settlement residents. The knowledge concerning grassland ecosystems is vanishing gradually because the related knowledge is no longer useful to the Mongols who are settled down or engaged in farming or other economic pursuits. The Mongolians in Inner Mongolia have been influenced by other cultures, e.g. in some areas Han Chinese words, including plant names, are more or less mixed up with the Inner Mongolians' spoken language. This may be leading to Mongols forgetting traditional botanical knowledge related to the language of plant folk names and classifications. Both artificial and natural factors lead to the degradation of the grassland and desertification. As a result, plant diversity that Mongolians traditionally named and used has decreased. The reduction of plant diversity may also lead to the extinction of the related knowledge of biodiversity. Thus it will be impossible to hand down to future generations. For this reason, collection and analysis of Mongolian plant folk names is extremely important.

Ethnobotanical surveys and analysis on wild plant folk names in the Mongolian language have been carried out since the 1990s [10-13]. However, studies on the relationship between Mongolian plant folk names and scientific names were only developed in the Ejina desert area, one of the Banners of western Inner Mongolia [14]. Therefore, ethnobotanical findings related to the Mongolian plant folk nomenclature are still fragmentary. Biodiversity has social, economic, ecological and ethical value. Understanding ecological functions of biodiversity, respecting the ethics and social importance of biodiversity, and the appropriate exploitation and use of biodiversity are the global issues facing biodiversity today. Scientists have paid close attention to the relationships between biodiversity and cultural diversity [15-18]. Mongolian traditional knowledge of biodiversity includes aspects of folk nomenclature, and traditional use and management of regional biodiversity. In this paper, in accordance with the ethnobotanical collections of the Mongolian folk names of wild plants in the Arhorchin National Nature Reserve, the relationship between folk names and scientific names are studied, and the structure of folk botanical nomenclature is also analyzed.

## Materials and methods

### Study area and ethnic group

**Figure 1:**
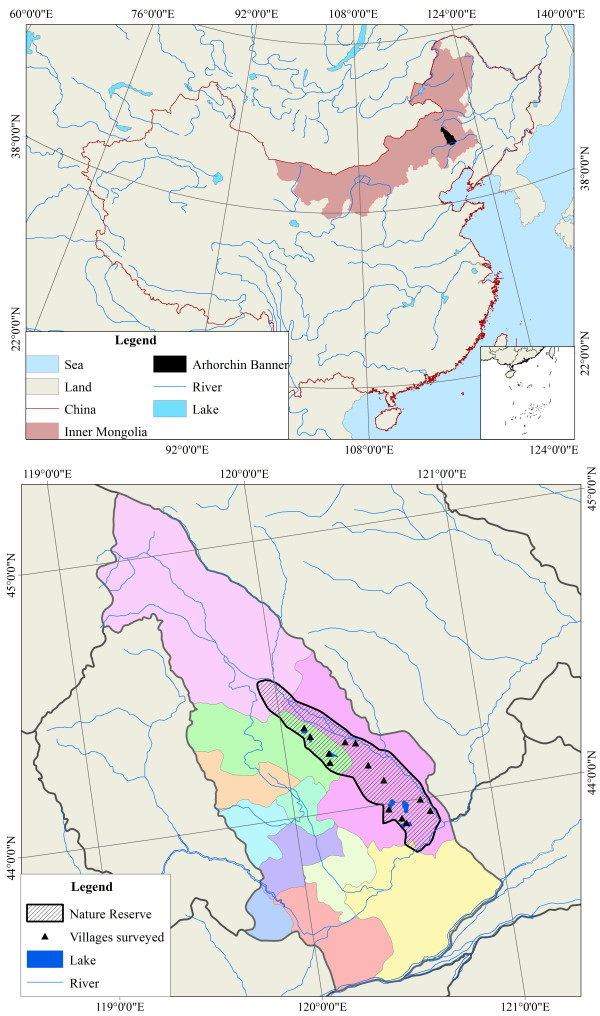
Study area and villages surveyed.

The Arhorchin National Nature Reserve is located in northeastern Inner Mongolia, China, at 43°48'30" - 44°28'31"N and 119°55'02"-120°41'27"E (Figure 1), with a land area of 1367.94 km^2^. The altitude ranges from 350 m to 400 m. This area has a temperate zone continental climate, with an average annual temperature of 6°C, and a mean rainfall of about 300 mm. The frost-free period in the area is from 130 to 140 days [19]. The Arhorchin National Nature Reserve is situated in the transitional zone area where mountain and hill of the Greater Hinggan Mountains transition to Horqin Sandy Land. There are many geomorphic types: sand, hills, river lowlands and lake. The zonal soil classification is chestnut soil. Other types of soil such as grey meadow soil is distributed in the swamp and ancient river area, and aeolian sandy soil is the soil type of sandy desertification land. This area belongs to the typical steppe sub-band of mesothermal steppe zone in the flora regional system of Inner Mongolia [20]. The vegetation form in the area is mainly shrub, steppe, meadow, swamp and aquatic vegetation. The steppe vegetation may be further classified as sparse forest steppe, typical steppe, and degenerated steppe. The meadow vegetation includes typical meadow, swamp meadow and saline meadow. According to related materials [21-26] and our investigations, about 350 species of vascular plants are distributed in this area.

Arhorchin, a Mongol word meaning northern archer, is the name of one of the descendent tribes of Genghis Khan’s brother Khasar. At the beginning of the Qing dynasty, the Arhorchin tribe and their occupied area was established as a Banner, and the tribe’s name was used as the Banner name [19]. In 1998, the Banner-level nature reserve was established. After two years, the Arhorchin Nature Reserve was approved by the People's Government of Inner Mongolia as a provincial level nature reserve. In 2004, the nature reserve was promoted to a national nature reserve, and was named “Arhorchin National Nature Reserve of Inner Mongolia”. As an open-ended type of nature reserve, there are more than 9500 local Mongol herdsmen’s pasture ranges involved in the nature reserve area. The local Mongol people still engaged in their traditional animal husbandry depend on wild plant resources in this area, and the local people still traditionally use local wild plants for various purposes.

### Methods

During 2008 to 2012, the authors have been to the nature reserve area 5 times. Field study was done in 13 villages of Agtanhua, Arbolag, Bayannuur, Burental, Chabintal, Chagantal, Dalha, Hagintal, Hunit, Nugustai, Shangxinmod, Tuburig, and Wuhercholuu, and 56 local Mongol herdsmen were interviewed as key informants, including 42 males and 14 females. Among them, 2 informants were over the age of 80, 15 informants at the age of 70–79, 17 informants at 60–69, 17 informants at 50–59, and 5 informants at 40–49. Ethnobotanical interviewing methods of free-listing and open-ended questionnaires were used [27-31]. Ethnobotanical interview and voucher specimen collection were carried out in two ways as local plant specimens were collected beforehand and then used in interviews, and local Mongol herdsmen were invited to the field and interviewed while collecting voucher specimens. Mongolian oral language was used as the working language and findings were originally recorded in Mongolian written language. Scientific names of plants were defined through collection and identification of voucher specimens by the methods of plant taxonomy. The voucher specimens were deposited in the Herbarium of the College of Life Science and Technology, Inner Mongolia Normal University.

## Results and discussion

A total of 146 folk names of local plants are recorded. Among them, more than 20 folk names are used two or three times for plant names by the local people. Based on the results of identifying the specimens, the folk names corresponded with 111 species, 1 subspecies, 7 varieties, 1 form, which belong to 42 families and 88 genera (Table 1). Among them, only one species of *Equisetum arvense* L. belonged to Pteridophyta and *Ephedra sinica* Stapf belonged to Gymnosperm. In the Table 1, plant families are arranged in accordance with the order of the Cronquist (Arthur John Cronquist, 1919–1992) system [32].

**Table 1:** The Correspondence between folk names of the Mongol herdsmen in Arhorchin national nature reserve and scientific classification

**Family**	**Folk names**	**Scientific names**
**Equisetaceae**	**sobrog ebes; onis ebes**	*Equisetum arvense* L.
**Ephedraceae**	**zhergen**	*Ephedra sinica* Stapf
**Ranunculaceae**	**ber checheg**	*Delphinium grandiflorum* L.
**Ulmaceae**	**delt**	*Ulmus macrocarpa* Hance
	**hailson mod**	*Ulmus pumila* L.
**Cannabaceae**	**olos; heerin olos**	*Cannabis sativa* f. *ruderalis* (Janisch.) Chu
**Moraceae**	**yilam**	*Morus mongolica* Schneid.
**Urticaceae**	**halgai**	*Urtica cannabina* L.
**Betulaceae**	**husu**	*Betula dahurica* Pall.
	**husu**	*Betula platyphylla* Suk.
**Chenopodiaceae**	**churgul**	*Agriophyllum pungens* (Vahl) Link ex A.Dietr
	**honin noil**	*Chenopodium acuminatum* Willd.
	**morin noil**	*Chenopodium album* L.
	**chagan turu**	*Kochia scoparia* (L.) Schrad.
	**hamhuul**	*Salsola collina* Pall.
	**hers**	*Suaeda glauca* (Bunge) Bunge
	**hers**	*Suaeda salsa* (L.) Pall.
**Amaranthaceae**	**arbai; wulaan bada**	*Amaranthus retroflexus* L.
**Portulacaceae**	**majinsai**	*Portulaca oleracea* L.
**Caryophyllaceae**	**baxig**	*Dianthus chinensis* var. *subulifolius* (Kitag) Ma
**Polygonaceae**	**bodorgan**	*Atraphaxis manshurica* Kitag.
	**gejige ebes**	*Polygonum aviculare* L.
	**ximeldegen**	*Polygonum divaricatum* L.
	**hurgan qihi**	*Polygonum lapathifolium* L.
**Plumbaginaceae**	**suun huar**	*Limonium bicolor* (Bunge) O.Kuntze
**Malvaceae**	**hima**	*Abutilon theophrasti* Medic
	**chagan huar**	*Hibiscus trionum* L.
	**taur nogo; eljigen taurai**	*Malva verticillata* L.
**Tamaricaceae**	**suhai**	*Tamarix chinensis* Lour.
**Salicaceae**	**wuliyas; honter mod**	*Populus davidiana* Dode
	**wuliyas; honter mod**	*Populus simonii* Carr.
	**wuliyas; honter mod**	*Populus simonii* Carr. var. *rotundifolia* S. C. Lu ex C. Wang et Tung
	**chagan bargas; yamaan bargas; xira bargas**	*Salix gordejevii* Y.L.Chang et Skv.
	**wuda**	*Salix matsudana* Koidz.
	**bor bargas**	*Salix microstachya* Turcz. var. *bordensis* (Nakai) C. F. Fang
**Brassicaceae**	**halun nogo**	*Lepidium apetalum* Willd.
**Rosaceae**	**taulain tangnai**	*Potentilla anserina* L.
	**taulain tangnai**	*Potentilla chinensis* Ser.
	**wulaan**	*Prunus humilis* Bunge
	**hargan; heerin guils**	*Prunus sibirica* L.
	**suden chai**	*Sanguisorba officinalis* L.
	**sheeber**	*Spiraea pubescens* Turcz.
**Fabaceae**	**togon shugur ebes**	*Astragalus melilotoides* Pall.
	**altagan**	*Caragana microphylla* Lam.
	**borchagt ebes**	*Glycine soja* Sieb.et Zucc.
	**xiher ebes**	*Glyeyrrhiza uralensis* Fisch.
	**hurbeg; hurbegen chai**	*Lespedeza davurica* (Laxm.) Schindl.
	**hurbeg; hurbegen chai**	*Lespedeza hedysaroides* (Pall.) Kitag.
	**dogol olos; dogol ebes**	*Sophora flavescens* Soland.
	**pojing ebes**	*Sphaerophysa salsula* (Pall.) DC.
**Thymelaeaceae**	**dalan turu**	*Stellera chamaejasme* L.
**Trapaceae**	**tumur zhanggu**	*Trapa japonica* Fler.
**Euphorbiaceae**	**tarnuu**	*Euphorbia esula* L.
	**malagan zhala**	*Euphorbia humifusa* Willd.
**Rhamnaceae**	**yaxil**	*Rhamnus arguta* Maxim.
	**yaxil**	*Rhamnus parvifolia* Bunge
**Vitaceae**	**heerin wujem**	*Ampelopsis aconitifolia* Bunge var. *glabra* Diels et Glig
**Aceraceae**	**hatu chagan**	*Acer truncatum* Bunge subsp. *mono* (Maxim.) E. Murr.
**Zygophyllaceae**	**tumer zhanggu**	*Tribulus terrestris* L.
**Geraniaceae**	**boh ebes**	*Erodium stephanianum* Willd.
**Apocynaceae**	**bargasen chai**	*Apocynum venetum* L.
**Asclepiadaceae**	**yamaan eber**	*Cynanchum chinense* R.Br.
	**temeen huh**	*Cynanchum thesioides* (Freyn)K.Schum.
	**nohon sheber**	*Periploca sepium* Bunge
**Solanaceae**	**oros zhanggu**	*Datura stramonium* L.
	**langdans**	*Hyoscyamus niger* L.
	**nohon wujem**	*Solanum nigrum* L.
**Convolvulaceae**	**hundagan huar**	*Convolvulus arvensis* L.
	**ayagan huar**	*Pharbitis purpurea* (L.) Voigt.
**Cuscutaceae**	**xar orongg**	*Cuscuta chinensis* Lam.
**Boraginaceae**	**nang zhanggu**	*Lappula heteracantha* (Ledeb.) Gurke
	**hor ebes**	*Messerschmidia sibirica* L. var. *angustior* (DC.) W. T. Wang
**Lamiaceae**	**durbelj ebes**	*Leonurus japonicus* Houtt.
	**durbelj ebes**	*Leonurus sibiricus* L.
	**huj ebes; heerin huajiao**	*Thymus serpyllum* L. var. *mongolicus* Ronn.
**Plantaginaceae**	**wuherin hel; chegulucai**	*Plantago asiatica* L.
	**wuherin hel; chegulucai**	*Plantago depressa* Willd.
	**wuherin hel; chegulucai**	*Plantago major* L.
**Asteraceae**	**morin xiranlj**	*Artemisia annua* L.
	**morin shabag**	*Artemisia brachyloba* Franch.
	**agi; altan agi**	*Artemisia frigida* Willd.
	**honin shabag**	*Artemisia halodendron* Turcz.
	**suih ebes;agi**	*Artemisia lavandulaefolia* DC.
	**erem**	*Artemisia sieversiana* Ehrhart ex Willd.
	**chonon haltar**	*Cirsium segetum* Bunge
	**auul ebes**	*Leontopodium leontopodioides* (Willd.) Beauv.
	**galuun chumchai**	*Mulgedium tataricum* (L.) DC.
	**tagsha**	*Neopallasia pectinata* (Pall.) Poljak.
	**hongolzuur**	*Serratula centauroides* L.
	**chumchai; yidere nogo;** **gaxiun nogo**	*Sonchus arvensis* L.
	**bobodeng**	*Taraxacum mongolicum* Hand.-Mazz.
	**honin zhanggu**	*Xanthium sibiricum* Patrin ex Widder
**Cyperaceae**	**zhuleg; zhuleg ebes**	*Carex duriuscula* C. A. Mey.
	**bumburen zheges**	*Scirpus tabernaemontani* Gmel.
	**gorbaljin zheges**	*Scirpus yagara* Ohwi
**Poaceae**	**deres**	*Achnatherum splendens* (Trin.) Nevski
	**mogailjin ebes**	*Agropyron cristatum* (L.) Gaertn.
	**chonon suul**	*Calamagrostis epigejos* (L.) Roth
	**chonon suul**	*Calamagrostis pseudophragmites* (Hall.f.) Koeler.
	**dagan suul; bolgan suul**	*Chloris virgata* Swartz.
	**hazhaar ebes**	*Cleistogenes squarrosa* (Trin.) Keng
	**tihan hul**	*Digitaria ischaemum* (Schreb.) Schreb. ex Muhl.
	**shuibaizi**	*Echinochloa crusgalli* (L.) Beauv.
	**shaag**	*Leymus chinensis* (Trin.)Tzvel.
	**hulus**	*Phragmites australis* (Cav.)Trin. ex Steud.
	**wulun chagan**	*Poa attenuata* Trin. ex Bunge
	**wulun chagan**	*Poa spondylodes* Trin. ex Bunge
	**shar tolgait**	*Setaria glauca* (L.) Beauv.
	**wurin suul**	*Setaria virdis* (L.) Beauv.
	**hilgan**	*Stipa grandis* P. Smirn.
**Typhaceae**	**odol; zhegs**	*Typha angustifolia* L.
	**habtgai odol; zhegs**	*Typha minima* Funck.
**Liliaceae**	**wumhi songgin**	*Allium condensatum* Turcz.
	**taan**	*Allium polyrhizum* Tirzc. ex. Regel
	**gogd; heerin gogd**	*Allium ramosum* L.
	**manggir**	*Allium senescens* L.
	**hereen nud**	*Asparagus dauricus* Fisch.ex Link
	**saralang huar**	*Lilium pumilum* DC.
**Iridaceae**	**chahirma**	*Iris lactea* Pall. var. *chinensis* (Fisch.) Koidz.
	**uhan sahal**	*Iris tenuifolia* Pall.

### The correspondence between plant folk names & scientific names

The plants folk names and scientific names (species) are not a simple one to one correspondence. It may be organized as below:

(a) *One to one correspondence* One folk name has correspondence with one scientific species. For example, the folk name zhergen with *Ephedra sinica* Stapf, ber checheg with *Delphinium grandiflorum* L., delt with *Ulmus macrocarpa* Hance, hailson mod with *Ulmus pumila* L. etc. In this case, a total of 78 single folk names has correspondence with 78 taxon, specifically as 72 species, 1 subspecies and 5 varieties, account for 53.42% in all the folk names including those used repeatedly.

(b) *Two or three to one correspondence* Two or three folk names have correspondence with only one scientific species. For example, sobrog ebes and onis ebes correspond with *Equisetum arvense* L., arbai and wulaan bada with *Amaranthus retroflexus* L.; habtgai odol and zhegs correspondence with *Typha minima* Funck.; chagan bargas, yamaan bargas, xira bargas correspondence with *Salix gordejevii* Y.L.Chang et Skv etc. In this case, those folk names corresponding with one scientific name are regarded as a folk synonym.

(c) *One to multitude correspondence* One folk name corresponds with two or more scientific species. For example, husu corresponds with *Betula dahurica* Pall. and *Betula platyphylla* Suk., hers with *Suaeda glauca* (Bunge) Bunge and *Suaeda salsa* (L.) Pall., yaxil with *Rhamnus arguta* Maxim. and *Rhamnus parvifolia* Bunge etc. In this case, there are 10 groups of folk names that have correspondence with 10 genera. Those folk names with correspondence with two or more scientific names are regarded as folk homonyms. Exceptional case was that folk name tumur zhanggu corresponds with *Trapa japonica* Fler. and Tribulus terrestris L. which belong to different families.

### Structure of the local Mongolian folk botanical nomenclature

A basic step in analyzing the structure of folk botanical nomenclature is to tell the difference between primary and secondary names and to distinguish between the various primary names [27]. According to the result of the linguistic analysis, the Mongolian folk names of wild plants in the Arhorchin National Nature Reserve are distinguished as primary names, secondary names and borrowed names.

#### *Primary names*

A primary name is considered to be 'semantically unitary' which means that it is a single expression, even if composed of more than one constituent. Many primary names have just a single constituent, and they belong to simple primary names, such as zhergen, delt, olos, yilam, halgai, husu, churgul, hamhuul, hers, bodorgan, hongolzuur, suhai, wuliyas, sheeber, altagan, yaxil, erem, ders, shaag, hulus, hilgan, taan, manggir etc. In the Mongolian language, these words are proper names which don’t have any other meanings. Other primary names are composed of more than one constituent which belongs to complex primary names. Complex primary names consist of two or three words. Some complex primary names include a word mod [tree] or ebes [grass] which indicates the life form, such as hailson mod, honter mod, sobrog ebes, gejige ebes, togon shugur ebes, borchagt ebes, xiher ebes etc. In this type of folk classification, a word mod or ebes serves as a taxon such as family or genus in scientific taxonomy. These types of names belong to productive complex primary names. Other complex primary names don't include a word to express a folk taxon, belonging to the unproductive complex primary name, such as eljigen taurai, dalan turu, malagan zhala, hatu chagan, yamaan eber, temeen huh, xar orongg etc.

#### *Secondary names*

Secondary names are formed from simple primary names by simply adding a modifier which further describes the plant. Among these types of names, simple primary names serve as a folk generic. For example, secondary names honin nuil (*Chenopodium acuminatum* Willd.) and morin nuil (*Chenopodium album* L.) are formed from the simple primary name nuil; bumburen zheges (*Scirpus tabernaemontani* Gmel.) and gorbaljin zheges (*Scirpus yagara* Ohwi) are formed from zheges; morin shabag (*Artemisia brachyloba* Franch.) and honin shabag (*Artemisia halodendron* Turcz.) are formed from shabag. A word nuil, zheges and shabag serves as a folk generic and equates to the scientific genus *Chenopodium*, *Scirpus* and *Artemisia*.

#### *Borrowed names*

Borrowed names are came from another language instead of Mongolian words. Among the folk names, such as majinsai, langdans, chegulucai, chumchai, bobodeng, shuibaizi from Han Chinese language, tarnuu and tagsha from Tibetan language, and baxig from Sanskrit. From this we observe that local Mongols have been under the influence of the Han Chinese traditional botanical culture or communication of different cultures for a long time.

The correspondence between plant folk names of the Mongol herdsmen in the Arhorchin National Nature Reserve and scientific names are similar to the different indigenous people in different countries. For example, the subdivisions of generics of wild plants are distinguishable through the Eipo peoples’ binomial nomenclature, e.g. *table, table kara, table nyana* for three different species of *Saurauia*[33]. The Mongol herdsmen in the Arhorchin National Nature Reserve use morin shabag and honin shabag for distinguish two different species of *Artemisia*. However, other species of *Artemisia* were used different names as agi, suih-ebes, erem by the the Mongol herdsmen in the Arhorchin National Nature Reserve. In the village of Theth, which has traditionally been inhabited by the Catholic Kelmendi and Shala tribes in the Northern Albanian Alps, *Chenopodium bonushenricus*, *Amarathus retroflexus* and *Amarathus lividus* represent what are known in ethnotaxonomy as prototypes. They are simply called nena, or nena e butë. The other members of this “nena group” (*Chenopodium album* and Rumex longifolius) are classified by the locals with the folk specifics nena e egër or nena elpjet [34]. In the Arhorchin National Nature Reserve, the Mongol herdsmen called *Chenopodium album* as morin noil, and called *Amarathus retroflexus* as arbai or wulaan bada. From this we can see that the classification of the same plant species is different between different ethnic groups.

### Special characteristic of the plant folk names of the Mongol herdsmen in the Arhorchin national nature reserve

Since herdsmen are most familiar with the physical characteristics of animals, when naming plants they easily transferred some of the applicable characteristics of animals to describe the plants. Hasbagan and Chen had discussed the fact that the Mongolians were accustomed to using the physical characteristics of animals in traditional plant nomenclature [10] As an important part of all Mongolians, the Mongol herdsmen in the Arhorchin National Nature Reserve also have this tradition. Typical kind is that the animal’s physical characteristics are used to describe features of the parts of plants. In this case, (an) animal’s beard, ear, eye, feet, hoof, horn, palate, tail, teat, and tongue are traditionally used to describe morphological characteristics of the parts of plants which (were) related (to) 15 species (Table 2).

**Table 2:** Nomenclature using animal physical characteristics to describe the morphological characteristics of the parts of plants

**Folk names**	**Meaning**	**Corresponding plants**	**Related part and morphological characteristics**
**chonon suul**	Wolf tail	*Calamagrostis epigejos*	Inflorescence shape
		*Calamagrostis pseudophragmites*	
**dagan suul**	Tail of 2 yrs horse	*Chloris virgata*	Inflorescence shape
**bogan suul**	Marten tail		
**eljigen taurai**	Donkey hoof	*Malva verticillata*	Leaf shape
**hereen nud**	Crow eye	*Asparagus dauricus*	Fruit shape
**hurgan qihi**	Lamb ear	*Polygonum lapathifolium*	Leaf shape
**taulain tangnai**	Rabbit palate	*Potentilla anserina*	Leaf shape
		*Potentilla chinensis*	
**temeen huh**	Camel teat	*Cynanchum thesioides*	Fruit shape
**tihan hul**	Chicken feet	*Digitaria ischaemum*	Inflorescence shape
**wuherin hel**	Bull tongue	*Plantago asiatica*	Leaf shape
		*Plantago depressa*	
		*Plantago major*	
**uhan sahal**	He-goat beard	*Iris tenuifolia*	Leaves shape
**yamaan eber**	Goat horn	*Cynanchum chinense*	Fruit shape

### Plant use information among the plant folk names

It can be seen that plant folk names are carrying important information about plant use in accordance with the meaning of the words of plant folk names (Table 3). Among them, local use information of five species and one variety of plants are showing no difference between folk names and the ethnobotanical interview.

**Table 3:** Plant use information among the plant folk names of the Mongol herdsmen in Arhorchin national nature reserve

**Scientific names**	**Folk names**	**Meaning**	**Local use**
*Amaranthus retroflexus*	**wulaan bada**	Red rice	—
*Apocynum venetum*	**bargasen chai**	Willow twig tea	Leaves as tea
*Lepidium apetalum*	**halun nogo**	Spicy vegetable	Seedlings as vegetable
*Astragalus melilotoides*	**togon shugur ebes**	Pan cleaning brush grass	—
*Lespedeza davurica*	**hurbegen chai**	**Hurbegen** tea	Stems, leaves and flower as tea
*Malva verticillata*	**taur nogo**	Peach vegetable	Tender leaves as vegetable
*Sanguisorba officinalis*	**suden chai**	**Suden** tea	Stems or roots as tea
*Sonchus arvensis*	**gaxiun nogo**	Bitter taste vegetable	Tender stems and leaves as vegetable
*Thymus serpyllum* var. *mongolicus*	**huj ebes;** **heerin huajiao**	Incense grass; wild Chinese prickly ash	Aboveground parts as condiment

According to the information from folk names, *Amaranthus retroflexus* and *Lepidium apetalum* may be edible plants. Unfortunately, ethnobotanical interviews have not found related materials in this area. However, according to related reports, seeds of *Amaranthus retroflexus* are used as grain by Arhorchin Mongol herdsmen in same area [35], and aboveground parts of *Astragalus melilotoides,* with leaves removed, were used to make pan brushes [36]. We think that this problem may be caused by two kinds of circumstances. It may be because our ethnobotanical interviews failed to cover more people in this area. Another possibility is the rapid disappearance of traditional knowledge on plant use. The reality is that the tradition of wild plant use is losing its practical utilization value with the economic development of this area. It's not necessary to collect and make pan brushes for herdsmen today, because it's so convenient to go to the store to buy pan cleaning brushes made of synthetic materials for the present day herdsmen.

### Comparison of plant folk names between the Mongol herdsmen in the Arhorchin national nature reserve with the Mongolians in Ejina desert area

In 2008, Khasbagan and Soyolt analyzed wild plants' folk names used by the Mongolians in the Ejina desert area. A total of 119 folk names of local plants were recorded; the folk names corresponded with 91 scientific species [14]. The statistic results showed that some 16 species and 1 variety are still the same despite different vegetation types between the two areas. Therefore, the comparisons must be carried out within the scope of these 16 species and 1 variety, others are incomparable. Folk names of 6 species were totally different between the two areas, they are also incomparable (Table 4). Among them, a folk name “hongolzuur” is a simple primary name, and it cannot be translated into any other language. From the folk names in the Table 4, it can be seen that the words which used as plant names and their meanings are completely different.

**Table 4:** Totally different folk names of plants among Arhorchin and Ejina

**Scientific names**	**Arhochin**	**Meanings**	**Ejina**	**Meanings**
*Cleistogenes squarrosa*	**hazhaar ebes**	Bit grass	**cagan ebes**	White grass
*Convolvulus arvensis*	**hundagan huar**	Winecup flower	**oryamug**	Entwine
*Limonium bicolor*	**suun huar**	Milk flower	**zer in deleng**	Breast of Mongolian gazelle
*Polygonum aviculare*	**gejige ebes**	Hair grass	**wuyet wulan**	Red has node
*Serratula centauroides*	**hongolzuur**	**——**	**gaxiun**	Bitter
*Sphaerophysa salsula*	**guzhe ebes**	Rumen grass	**porqigenur; honht ebes**	Crackling; grass has bell

Among the other 10 species and 1 variety, folk name taan of *Allium polyrhizum* was completely the same, comparison will not be needed. In the remaining 9 species and 1 variety, the relationship between the plant folk names from two areas are complicated. Firstly, *Agriophyllum pungens* was called churgul in Arhorchin and called sulker in Ejina. In modern Mongolian language, churgul and sulker are called [culihir]. The authors considered that churgul and sulker represented Mongolian dialects in different regions. *Glyeyrrhiza uralensis* was called xiher ebes in Arhorchin, and called xiker buyaa in Ejina. The first word of the name xiher and xiker were a different pronunciation of the same word [sihir] which means sugar in modern Mongolian language. In the name of xiher ebes, the second word [ebes] means grass, so the whole name means ‘sweet grass’. In Ejina, a folk generic buyaa usually is used to name the plants which have fleshy roots [14]. Secondly, at least one name is the same between the two areas. *Achnatherum splendens* has only one name, deres, in Arhorchin, but has two names, deres and tongge, in Ejina. The name deres was the same in both areas. *Iris lactea* var. *chinensis* has only one name, chahirma, in Arhorchin, but has two names, cakildag and cakirma, in Ejina. The name cakirma was the same in both areas. *Artemisia frigida* has two names agi and altan agi in Arhorchin, and has two other names xiaralji and agi in Ejina. The name agi was the same in both areas. *Phragmites australis* has only one name hulus in Arhorchin, but has six names, hulus, acamag, shagxig hulus, shaorag hulus, hana hulus, and ajirgan hana in Ejina. Only one name hulus was the same between two area. Thirdly, the Mongol primary name noil of *Chenopodium* was used in different ways between the two areas. In Arhorchin, the Mongol folk name of *Chenopodium acuminatum* and *Chenopodium album* was called honin noil and morin noil, which belong to the secondary name based on the primary name noil. However, the primary name noil is used as a species name in Ejina. Fourthly, *Sonchus arvensis* has three different names in Arhorchin and only one name in Ejina. They both have the same word, gaxiun, composing the folk name gaxiun nogo and gaxiun ebes. Using the word gaxiun, meaning ‘bitter’, the whole name can be translate into ‘bitter vegetable’ and ‘bitter grass’. *Tribulus terrestris* was called tumer zhanggu in Arhorchin and called yamaan zhanggu in Ejina. Both have the same word zhanggu. The word zhanggu usually is used to name the plants which have prickly fruits by local Mongols, such as the species of *Lappula*, *Tribulus*, *Xanthium* etc.

Some people may pose the question of why do different groups of the same nationality use different names for plants? The authors think that the answers to this question related to the history and status of Mongolian tribes. Mongolian traditional culture formed under certain natural environment, economic and social conditions. Different tribes of the Mongolian people living in different regions, in different natural environment conditions, in different economic and social development stage, which caused them to have certain differences in language and culture. The difference of their traditional botanical knowledge and culture also caused this reason. Arhorchin Mongolians belongs to the Mongolian Horchin tribe, from the area of Ergun (Argun) River and Hulun Buir Lake moved to the present place for more than 400 years. However, the majority of Ejina Mongolians belongs to the Mongolian Oirat tribe, from the area of Volga River Basin moved to the present place for more than 300 years. The distance between Arhorchin Banner and Ejina Banner is about 2000 kilometers. They hardly have contact with each other, they did not have the opportunity and needs to exchange traditional botanical knowledge. This confirms the regional characteristics of the traditional botanical knowledge.

## Conclusion

The Mongol herdsmen in the Arhorchin National Nature Reserve were capable of naming 120 species including infraspecific taxa in their own language-Mongol language. In the corresponding rate between plant folk names and scientific names, there are 146 folk names that have correspondence with 120 scientific names of plant species and infraspecific taxa, yielding a computational correspondence of 82.19%. The authors think that this corresponding rate was quite high, the local Mongol herdsmen’s plant nomenclature have very high accuracy. It also can be considered as one of the typical case that the level of consistency between scientific knowledge and traditional knowledge in botanical nomenclature.

Structurally, folk names of the Mongol herdsmen in the Arhorchin National Nature Reserve were classified as primary names, secondary names and borrowed name. Primary names have the most cultural significance in the plant folk names. However, Secondary names indicated the existence of folk generic, and have important meanings for folk classification. Borrowed names indicated that local Mongols have had cultural exchanges with other ethnic groups for a long time.

Special characteristic of the local Mongol herdsmen’s wild plant nomenclature was undoubtedly focused on physical characteristics of animals, particularly familiar domestic animals like cow, sheep, goat, horse, camel, and steppe wild animals like the wolf, marten, rabbit, crow etc. It can be inferred that the local Mongol herdsmen’s traditional botanical nomenclature is closely related to their traditional animal husbandry and Inner Asian steppe land environment.

We have seen plant folk names carrying important information about plant use from this case study. In this sense, plant folk names are not only a code to distinguish between different plant species, but also a kind of culture rich in a deep knowledge concerning nature.

The results of comparative analysis of the Mongol herdsmen in the Arhorchin National Nature Reserve and the Mongolians in the Ejina desert area shows that there are some similarities, as well as many differences in language and in the structure of plant names. From this we can infer that Mongolian culture, in terms of plant nomenclature, have characteristics of diversity between the different regions and different tribes.

Folk botanical nomenclature and classification of the Mongol herdsmen in the Arhorchin National Nature Reserve is an important part of their natural culture. This type of knowledge and culture has a great effect on their adaptation to the environment, utilization of plant resources and traditional biodiversity management on the community level.

Why the establishment of a nature reserve in that place? Because the ecosystem and its biodiversity has been preserved so well. Then, Why is the ecosystem there so good? The land of the Arhorchin National Nature Reserve today used to be the grazing areas of the herdsmen in the near past. In this area, local herdsmen were engaged in animal husbandry from generation to generation. However, the environment here is not damaged yet, the ecosystem there is still very healthy. All of these are due to herdsmen, past and the present. It has been preserved because of the herdsmen’s mode of production, model of life, and traditional culture concerning the natural environment. Luckily, the herdsmen were not moved from this area after the establishment of the nature reserve. The local Mongol people still traditionally use local wild plants for various purposes, and actively cooperate with the administration bureau of the nature reserve.

## Competing interest

The authors declare that they have no competing interest.

## Authors’ contributions

The fieldwork for data collection were conducted by S, G, Y, Wand K. Identification of voucher specimens were by S, GL and K. Data analysis and manuscript preparation were by S and K. All authors read and approved the final manuscript.
